# Pharmacokinetics, Safety, and Tolerability of Single and Multiple Doses of Isavuconazonium Sulfate in Healthy Adult Japanese Subjects

**DOI:** 10.1002/cpdd.1079

**Published:** 2022-02-21

**Authors:** Shinichiro Shirae, Atsushi Ose, Yuji Kumagai

**Affiliations:** ^1^ Development Planning, Clinical Development Center Asahi Kasei Pharma Corporation Tokyo Japan; ^2^ Kitasato University Hospital Kanagawa Japan

**Keywords:** isavuconazole, isavuconazonium sulfate, pharmacokinetics, safety, tolerability

## Abstract

Isavuconazonium sulfate is the water‐soluble prodrug of the novel, broad‐spectrum, triazole antifungal agent isavuconazole. This was a first‐in‐Japanese study assessing the pharmacokinetics, safety, and tolerability of isavuconazonium sulfate. The study was conducted in 2 parts: part 1 (single ascending dose; 100‐, 200‐, and 400‐mg equivalent of isavuconazole oral or intravenous administration); and part 2 (multiple doses for 16 days; 200‐mg equivalent of isavuconazole oral or intravenous administration; once‐daily administration with a loading regimen every 8 hours for the first 48 hours). A total of 60 and 16 subjects were randomized in part 1 and part 2, respectively. Observed clearance was lower in this study compared to what was previously reported in predominantly White populations and similar to clearance in non‐Japanese Asian populations. The range of the plasma isavuconazole concentration in this study was within the range of the pivotal phase 3 study, with no relationship between isavuconazole exposure and either efficacy or safety. There were no serious adverse events, and all reported treatment‐emergent adverse events were of mild intensity. This study confirmed that isavuconazonium sulfate was safe and well tolerated in healthy adult Japanese subjects.

Isavuconazonium sulfate (BAL8557) is a water‐soluble, triazole, antifungal agent that is rapidly hydrolyzed by plasma esterases to the active moiety, isavuconazole (BAL4815).^1^ Isavuconazole inhibits lanosterol 14α‐demethylase, a microsomal cytochrome P450 (CYP) enzyme essential for ergosterol biosynthesis in fungi.^2^ Isavuconazole is metabolized mainly by CYP3A4 and CYP3A5, and urinary recovery of isavuconazole was <0.4%.^1^, ^3^ Isavuconazonium sulfate is available in cyclodextrin‐free intravenous (IV) and oral (PO) formulations.^4^ The bioavailability of isavuconazole was reported as 98%, and the pharmacokinetic (PK) profile has been demonstrated to be similar between the PO and IV formulations of isavuconazonium sulfate, allowing for switching between formulations without dose adjustment.^1^, ^5^ Additionally, no food effect was reported with the PO formulation.^5^, ^6^ Similarly, no effect on clinical utility was reported in renally impaired patients.^6^, ^7^

Isavuconazonium sulfate has been used for the treatment of invasive aspergillosis and invasive mucormycosis in the United States and European countries.^6^ The approved dosage regimen is a 200‐mg equivalent of isavuconazole every 8 hours for 6 doses (48 hours) as a loading dose followed by a once‐daily 200‐mg maintenance dose. A phase 3 study of isavuconazonium sulfate has been conducted in Japan for regulatory approval in Japan (ClinicalTrials.gov Identifier: NCT03471988).

It is possible that ethnic factors affect a drug's safety, efficacy, dosage, and dose regimen.^8^ During isavuconazonium sulfate development, clinical studies were conducted in multiple regions, including the Asian region, such as China and Korea.^9^, ^10^, ^11^ A population pharmacokinetics (PPK) analysis using healthy subjects and patients reported race as a covariate on clearance (CL).^12^ The PPK analysis showed that the CL value estimated for a non‐Japanese Asian population (1.5 L/h) was ≈36% lower than the one for a predominantly White population (2.4 L/h).^12^ Another PPK analysis reported a similar result. Healthy Chinese subjects were found to have, on average, a 40% lower CL compared to healthy Western subjects (1.66 L/h for Chinese subjects compared to 2.57 L/h for Western subjects) and, therefore, an ≈50% higher area under the plasma concentration–time curve (AUC) than Western subjects.^13^

Although lower CL was observed in Asian populations, 2 exposure‐response analyses suggested no exposure‐response relationship for efficacy or safety within the concentration range achieved in the pivotal phase 3 study (SECURE study) in which patients of several races, including Asian, were enrolled.^14^, ^15^

Although the impact of ethnic factors has been previously assessed,^12^, ^13^, ^14^ no study has involved Japanese subjects. Therefore, this first‐in‐Japanese phase 1 study was conducted to investigate the pharmacokinetics, safety, and tolerability of isavuconazonium sulfate in a Japanese population.

## Methods

### Study Design

This was the first phase 1 study of isavuconazonium sulfate in Japan and was conducted in 2 parts: a randomized, double‐blind, placebo‐controlled, single‐ascending‐dose part (part 1) and an open‐label, noncontrolled, multiple‐dose part (part 2) in healthy Japanese male subjects. The dosage regimen is summarized in Table 1.

**Table 1:** Dosage Regimen

Part	Dose^a^	Administration Period	Route of Administration	Infusion Time	Treatment Arm	Number of Subjects
Part 1 (single dose)	Cohort 1: 100 mg	1 day	PO	N/A	Active	8
					Placebo	2
			IV	1 hour	Active	8
					Placebo	2
	Cohort 2: 200 mg	1 day	PO	N/A	Active	8
					Placebo	2
			IV	1 hour	Active	8
					Placebo	2
	Cohort 3: 400 mg	1 day	PO	N/A	Active	8
					Placebo	2
			IV	2 hours	Active	8
					Placebo	2
Part 2 (multiple dose)	200 mg	16 days	PO	N/A	Active	8
			IV	1 hour	Active	8

IV, intravenous; N/A, not applicable; PO, oral.

a Dose corresponds to milligram equivalent of isavuconazole.

In part 1, subjects were divided into 3 cohorts given different single doses of isavuconazonium sulfate. Each cohort was further divided by the route of administration (PO and IV). For each route of administration per cohort, 10 male subjects were randomized to receive a single dose of isavuconazonium sulfate (8 subjects) or placebo (2 subjects). Isavuconazonium sulfate doses given were 100‐, 200‐, or 400‐mg equivalent of isavuconazole (corresponding to 186.3, 372.6, or 745.2 mg of isavuconazonium sulfate). In this article, dosing information is expressed as the isavuconazole equivalent of the prodrug isavuconazonium sulfate.

In part 2, 8 male subjects each received multiple PO or IV administrations of 200‐mg equivalent of isavuconazole, respectively. The subjects received a loading dose of 200‐mg equivalent of isavuconazole every 8 hours for the first 48 hours (ie, days 1 and 2), followed by a daily maintenance dose of 200‐mg equivalent of isavuconazole up to day 16.

For PO administration, isavuconazonium sulfate was given as capsules containing a 100‐mg equivalent of isavuconazole, administered together with 150 mL of water. For IV administration, isavuconazonium sulfate in 250 mL of saline was infused. An infusion time period of 1 hour was selected for doses of 100‐ and 200‐mg equivalent of isavuconazole, whereas a 2‐hour infusion time was selected for the dose of 400‐mg equivalent of isavuconazole to reduce infusion‐related reactions caused by high infusion speed. This study was conducted in accordance with the International Conference on Harmonisation E6 Good Clinical Practice and the principles expressed in the Declaration of Helsinki. The study was performed at Clinical Research Hospital Tokyo, and the study protocol was reviewed and approved by an institutional review board (IHL Shinagawa East One Medical Clinic IRB, Tokyo, Japan). Written, informed consent was obtained from each subject before any study‐related procedures were performed.

### Study Population

Healthy Japanese male volunteers aged 20 to 44 years capable of sufficiently comprehending a description of the study and competent to give informed consent were included. Healthy subjects were defined as having no clinically relevant abnormalities identified by medical history, physical examination, vital signs, 12‐lead electrocardiogram (ECG), and clinical laboratory tests. Key exclusion criteria included body weight <50.0 kg; body mass index <18.5 or >25.0 kg/m^2^; or evidence of liver disease or liver injury as indicated by abnormal liver tests, such as alanine aminotransferase, aspartate aminotransferase, or γ‐glutamyl transferase levels exceeding the upper limit of normal. Subjects with a medical history of short QT syndrome or QT interval corrected for heart rate using Fridericia's formula <360 milliseconds were also excluded, because dose‐related shortening of the corrected QT interval was reported, and isavuconazonium sulfate is contraindicated in patients with familial short QT syndrome.^16^

### Subject Disposition and Demographics

In part 1, 60 subjects were enrolled, and 58 of them completed the study. Two subjects, 1 subject receiving IV administration of a 100‐mg equivalent of isavuconazole and 1 subject receiving IV administration of placebo, discontinued the study. The discontinuations of the study by these 2 subjects were the subjects’ wishes. All subjects were evaluable for the safety analyses. Of the 48 subjects receiving isavuconazole, 46 were evaluable for PK analyses, including the discontinued subject receiving IV administration of 100‐mg equivalent of isavuconazole for whom PK samples for 10 days following the start of administration of isavuconazonium sulfate were available. Two subjects receiving IV administration of 200‐mg equivalent of isavuconazole were excluded from the PK analyses because of errors encountered during isavuconazonium sulfate administration, such as infusion site extravasation. In part 2, 16 subjects were enrolled, and all subjects completed the study. All subjects were evaluable for safety and PK analyses.

Demographics and baseline characteristics are summarized in Table 2; age, height, weight, and body mass index were similar between the groups. In part 1, the mean ages of the isavuconazonium sulfate and placebo groups were from 24.6 to 31.8 years and from 26.3 to 27.3 years, respectively. The mean weights in the isavuconazonium sulfate and placebo groups were from 58.4 to 62.1 kg and from 61.1 to 62.3 kg, respectively. In part 2, the mean ages and weights were from 27.9 to 33.1 years and from 58.1 to 60.4 kg, respectively.

**Table 2:** Summary of Demographics

			Isavuconazonium Sulfate^a^							
			Single Administration						Multiple Administrations	
	Placebo		100 mg		200 mg		400 mg		200 mg	
	PO	IV	PO	IV	PO	IV	PO	IV	PO	IV
	(N = 6)	(N = 6)	(N = 8)	(N = 8)	(N = 8)	(N = 8)	(N = 8)	(N = 8)	(N = 8)	(N = 8)
Age, y, mean (SD)	26.3 (5.1)	27.3 (5.7)	31.8 (5.4)	30.9 (6.3)	28.5 (5.0)	29.0 (4.4)	24.6 (3.2)	29.8 (7.4)	33.1 (5.4)	27.9 (4.2)
Height, cm, mean (SD)	170.6 (4.3)	170.9 (3.6)	169.3 (4.1)	171.2 (3.7)	171.0 (5.0)	165.9 (2.1)	171.8 (4.0)	171.3 (3.6)	168.9 (2.5)	171.5 (4.1)
Weight, kg, mean (SD)	61.1 (6.4)	62.3 (5.2)	58.6 (4.9)	62.1 (5.5)	60.9 (5.2)	58.4 (4.2)	61.4 (2.8)	60.9 (2.8)	58.1 (5.8)	60.4 (4.8)
BMI, kg/m^2^, mean (SD)	21.0 (1.8)	21.3 (1.7)	20.4 (1.0)	21.2 (1.5)	20.8 (1.8)	21.2 (1.5)	20.9 (1.2)	20.8 (1.3)	20.4 (1.9)	20.6 (1.9)

BMI, body mass index; IV, intravenous; PO, oral; SD, standard deviation.

a Dose corresponds to milligram equivalent of isavuconazole.

### Bioanalytical Methods for PK Samples

Concentrations of isavuconazole in plasma were measured using a validated liquid chromatography coupled with tandem mass spectrometry method.^11^ Isavuconazole levels were measured in all plasma samples taken from subjects who received isavuconazonium sulfate. The lower limit of quantification was 5 ng/mL.

### Pharmacokinetic Assessments

During part 1, plasma samples for the measurement of isavuconazole concentration were taken at the following time points: before dosing; at 30 and 45 minutes; and 1, 1.5, 2, 3, 4, 5, 6, 8, 12, 24, 36, 48, 72, 96, 144, 240, 336, 432, and 480 hours after the start of isavuconazonium sulfate administration (PO or IV) on day 1. During part 2, plasma samples were taken at every predose point. In addition, plasma samples were taken at 30 and 45 minutes and 1, 1.5, 2, 3, 4, 5, 6, 8, 12, 24, 36, 48, 72, 96, 144, 240, 336, 432, and 480 hours after the start of isavuconazonium sulfate administration (PO or IV) on day 16. After collection, samples were processed immediately and stored at −80°C until shipment to the central research laboratory.

PK parameters were calculated by noncompartmental analysis using WinNonlin Version 7.0 (Pharsight Corporation, Mountain View, California). Calculations were based on the actual sampling times recorded during the study. Although subjects who received PO and IV administration are different, bioavailability was calculated using the mean AUC from time 0 to infinity (AUC_inf_) values for each cohort. The dose proportionality of AUC from time 0 to the last quantifiable measurement (AUC_last_) following single administration was assessed using linear regression with the following power model (*log* [PK parameter] = *a* + *b* × *log* [dose] + *error*).

### Safety Assessment

Safety and tolerability assessments included physical examination, vital signs (blood pressure, heart rate, and axillary temperature), 12‐lead ECG, laboratory tests (hematology, biochemistry, and urinalysis), and adverse events. The safety analysis set included all subjects who received at least 1 dose of isavuconazonium sulfate or matching placebo.

## Results

### Pharmacokinetics of Isavuconazole After Single Administration (Part 1)

**Figure 1:**
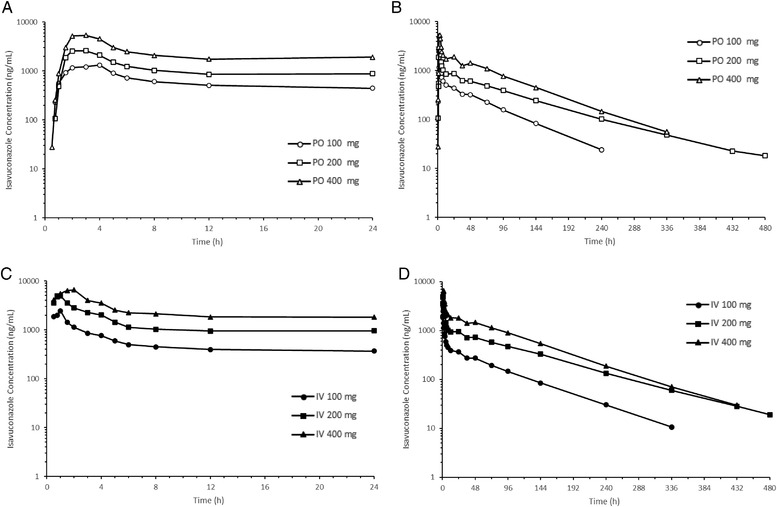
Mean plasma isavuconazole concentration–time profile after single administration of isavuconazonium sulfate. (A, B) Mean plasma concentration–time profile after single oral administration of isavuconazonium sulfate at 24 hours (A) and up to 480 hours (B). (C, D) Mean plasma concentration–time profile after single intravenous administration of isavuconazonium sulfate at 24 hours (C) and up to 480 hours (D). PO 100 mg, oral administration of 100‐mg equivalent of isavuconazole; PO 200 mg, oral administration of 200‐mg equivalent of isavuconazole; PO 400 mg, oral administration of 400‐mg equivalent of isavuconazole; IV 100 mg, intravenous administration of 100‐mg equivalent of isavuconazole; IV 200 mg, intravenous administration of 200‐mg equivalent of isavuconazole; IV 400 mg, intravenous administration of 400 mg equivalent of isavuconazole. IV, intravenous; PO, oral.

The mean plasma concentration–time profiles of isavuconazole after single PO and IV administration of isavuconazonium sulfate are presented in Figure 1. Similar plasma concentration–time profiles of isavuconazole were observed between single PO and IV administration of isavuconazonium sulfate (Figure S1). After single PO and IV administration, plasma concentrations of isavuconazole increased rapidly and then decreased gradually. Plasma concentrations of isavuconazole increased as the dose increased.

PK parameters of isavuconazole after single PO and IV administrations of isavuconazole are presented in Table 3. After PO and IV administrations of isavuconazonium sulfate, maximum plasma concentration (C_max_), AUC_last_, and AUC_inf_ increased as the dose increased. In the dose proportionality assessment, the slope estimates for AUC_last_ were 1.106 (95%CI, 0.866‐1.346) for PO administration and 1.217 (95%CI, 1.014‐1.419) for IV administration. For PO administration, time to maximum concentration (t_max_), terminal elimination half‐life (t_1/2_), and apparent CL were similar across the dose levels. For IV administration, t_max_ occurred when the infusion was ended, and t_1/2_, CL, and volume of distribution were similar across the dose levels. C_max_ tended to be higher with IV than with PO administration, and t_max_ was greater with PO than with IV administration. The other PK parameters were similar between PO and IV administrations. Bioavailability for 100‐, 200‐, and 400‐mg equivalent of isavuconazole were 106.5%, 82.0%, and 89.1%, respectively.

**Table 3:** Summary of Pharmacokinetic Parameters (Part 1: Single‐Ascending‐Dose Part)

	Dose^a^					
	Oral Administration			Intravenous Administration		
	100 mg (N = 8)	200 mg (N = 8)	400 mg (N = 8)	100 mg (N = 8)	200 mg (N = 6)	400 mg (N = 8)
C_max,_ ng/mL, mean (SD)	1657 (434)	2933 (578)	5844 (989)	2466 (138)	5382 (768)	6690 (596)
AUC_last_, ng·h/mL, mean (SD)	46 130 (19 023)	107 802 (28 229)	208 614 (61 055)	42 599 (7627)	132 573 (38 447)	233 514 (53 640)
AUC_inf_, ng·h/mL, mean (SD)	46 803 (19 033)	110 737 (28 800)	210 510 (62 149)	43 956 (7424)	135 004 (39 941)	236 235 (56 142)
t_max_, h, median (range)	2.5 (1.0‐4.0)	2.5 (2.0‐3.0)	2.0 (2.0‐4.0)	1.0 (1.0‐1.0)	0.9 (0.8‐1.0)	2.0 (1.5‐2.5)
t_1/2_, h, mean (SD)	51.3 (17.6)	83.9 (30.7)	56.7 (21.4)	67.4 (23.9)	76.0 (15.9)	66.0 (19.8)
CL,^b^ mL/h, mean (SD)	2396 (772)	1949 (661)	2063 (648)	2329 (372)	1597 (474)	1808 (578)
V_d_,^c^ mL, mean (SD)	N/C	N/C	N/C	222 415 (74 533)	168 554 (40 328)	161 242 (22 745)
F, %, mean	106.5	82.0	89.1	N/A	N/A	N/A

AUC_inf_, area under the plasma concentration–time curve from time 0 to infinity; AUC_last_, area under the plasma concentration–time curve from time 0 to the last quantifiable measurement; C_max_, maximum plasma concentration; CL, clearance; F, bioavailability; N/A, not applicable; N/C, not calculated; SD, standard deviation; t_1/2_, terminal elimination half‐life; V_d_, volume of distribution.

a Dose corresponds to milligram equivalent of isavuconazole.

b CL corresponds to CL/F after oral administration.

c V_d_ was calculated by the area method.

After PO administration of 200‐mg equivalent of isavuconazole, the mean C_max_, AUC_last_, AUC_inf_, and apparent CL were 2933 ng/mL, 107 802 ng·h/mL, 110 737 ng·h/mL, and 1949 mL/h, respectively. After IV administration of 200 mg equivalent of isavuconazole, the mean C_max_, AUC_last_, AUC_inf_, CL, and volume of distribution were 5382 ng/mL, 132 573 ng·h/mL, 135 004 ng·h/mL, 1597 mL/h, and 168 554 mL, respectively.

### Pharmacokinetics of Isavuconazole After Multiple Administrations (Part 2)

**Figure 2:**
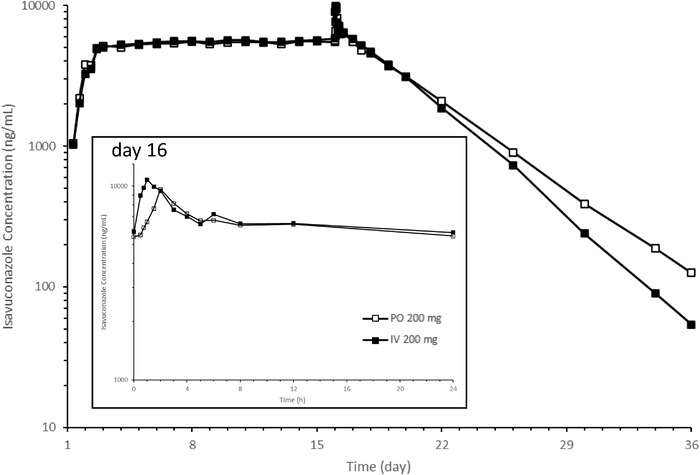
Mean plasma isavuconazole concentration–time profile after multiple administrations of 200‐mg equivalent of isavuconazole. The inset shows an expanded plot from before dosing to 24 hours on day 16. PO 200 mg, oral administration of 200‐mg equivalent of isavuconazole; IV 200 mg, intravenous administration of 200‐mg equivalent of isavuconazole. IV, intravenous; PO, oral.

The mean plasma concentration–time profiles of isavuconazole after multiple PO and IV administrations of 200‐mg equivalent of isavuconazole are presented in Figure 2. Similar plasma concentration–time profiles were observed between multiple PO and IV administrations. Trough concentrations of isavuconazole increased gradually during the loading dose period up to day 3, and similar trough concentrations were maintained on days 3 to 16. After the last PO and IV administrations, plasma concentration reached its maximum and then decreased gradually.

PK parameters of isavuconazole after multiple PO and IV administrations of 200‐mg equivalent of isavuconazole are presented in Table 4. The pharmacokinetic parameter values were similar between multiple PO and IV administrations. For multiple PO administrations, the mean trough concentration was 5479 ng/mL, and the mean C_max_ and AUC at steady state over the dosing interval after the last oral administration were 9720 ng/mL and 153 108 ng·h/mL, respectively. For multiple IV administrations, the mean trough concentration was 5817 ng/mL, and the mean C_max_ and AUC at steady state over the dosing interval after the last IV administration were 10 970 ng/mL and 160 014 ng·h/mL, respectively.

**Table 4:** Summary of Pharmacokinetic Parameters (Part 2: Multiple‐Dose Part)

	Dose^a^	
	Oral Administration	Intravenous Administration
	200 mg (N = 8)	200 mg (N = 8)
C_trough_,^b^ ng/mL, mean (range) SD	5479 (3508‐7409) 1456	5817 (3303‐9839) 2033
C_max_, ng/mL, mean (SD)	9720 (2422)	10 970 (2259)
AUC_τ,ss_, ng·h/mL, mean (SD)	153 108 (38 248)	160 014 (48 867)
t_max_, h, median (range)	2.0 (2.0‐3.0)	1.0 (0.8‐2.0)

AUC _τ,ss_, area under the plasma concentration–time curve over the dosing period at steady state; C_max_, maximum plasma concentration; C_trough_, trough concentration; SD, standard deviation; t_max_, time to maximum concentration.

a Dose corresponds to milligram equivalent of isavuconazole.

b Concentration before dosing on day 16.

### Safety

Isavuconazonium sulfate was well tolerated. There were no serious adverse events, and no subjects were withdrawn from the study due to treatment‐emergent adverse events (TEAEs).

In part 1, of the 48 subjects receiving single PO or IV administration of isavuconazonium sulfate, 8 subjects reported 9 TEAEs. Of 12 subjects receiving placebo, 1 subject reported 1 TEAE. All TEAEs were mild. The most frequently reported TEAEs were infusion site extravasation and increased alanine aminotransferase, which were reported in 2 subjects. Hematuria in 1 subject was reported as a drug‐related TEAE. No ECG abnormalities were reported, and there were no clinically relevant changes in vital signs.

In part 2, of the 16 subjects receiving multiple PO or IV administrations of isavuconazonium sulfate, 9 subjects reported 11 TEAEs. All TEAEs were mild. The most frequently reported TEAEs were infusion site reaction in 7 subjects who received multiple IV administrations. Infusion site reactions in 7 subjects and headaches in 2 subjects were reported as drug‐related TEAEs. No ECG abnormalities were reported, and there were no clinically relevant changes in vital signs.

## Discussion

This was the first study to assess the pharmacokinetics, safety, and tolerability of isavuconazonium sulfate in healthy Japanese male adults. The PK results in this study were mostly consistent with the findings reported in non‐Japanese populations: low systemic clearance, accumulation after multiple doses, bioavailability of 82.0% to 106.5% in this study, and comparable PK characteristics between PO and IV administrations.^6^

In the present study, systemic exposure (C_max_ and AUC) of isavuconazole after single PO administration of 200‐mg equivalent of isavuconazole was compared with that of a previously reported study whose subjects were predominantly healthy White male volunteers.^1^ In the present study, mean C_max_, AUC_last_, and AUC_inf_ after single PO administration of 200‐mg equivalent of isavuconazole were 2933 ng/mL, 107 802 ng·h/mL, and 110 737 ng·h/mL, respectively; the corresponding values reported in the previous study were 2.59 μg/mL, 77.7 μg·h/mL, and 78.5 μg·h/mL, respectively.^1^ Mean C_max_ values were similar between the studies, but AUC was higher in the present study than in the previous study. This observed difference in AUC is thought to be due to the difference in CL. Mean apparent CL was lower in the present study (1949 mL/h) than in the previous study (2.59 L/h).^1^

Lower CL than in a predominantly White population was also found with IV administration. CL after single IV administration of 200‐mg equivalent of isavuconazole was 1597 ± 474 mL/h in the present study, lower than the previously reported population mean estimate of CL in a predominantly White population (2.4 L/h) and similar to the one in a non‐Japanese Asian population (1.5 L/h).^12^ Therefore, the Japanese population may have lower CL than White populations, and similar CL to non‐Japanese Asian populations.

The reason for the lower CL values in the present study has not been established. The mean age in the present study was comparable to that of the previous study. Genetic polymorphism of isavuconazole metabolism is unlikely to explain the observed difference. PK differences caused by genetic polymorphism of CYP2C9, CYP2C19, and CYP2D6 are well established,^17^ but isavuconazole is metabolized mainly by CYP3A4 and CYP3A5.^3^ Lower clearance in Asian populations compared to White populations has been reported for some CYP3A substrate drugs, even after normalization for body size (weight or body surface area).^18^, ^19^ The reason for the difference is not known, but polymorphism of CYP3As has not been suggested as the reason for the lower clearance of the CYP3A substrate drugs.^18^, ^19^ The mean weight in the oral 200‐mg equivalent of isavuconazole group was 60.9 kg in the present study, which was ≈20% lower than in the previous study (73.9 kg). This lower weight may explain a part of the lower CL in this present study. However, because previous PPK analyses of isavuconazole showed that weight was not a significant covariate on CL,^11^, ^12^, ^13^ effect of weight on CL may be limited. Further investigation, such as a PPK analysis in the Japanese population, may be useful to confirm the effect of weight on CL.

Although lower CL was seen in the present study, the range of the observed trough concentration at steady state in part 2 of the present study (3303‐9838 ng/mL) was within the range of the pivotal phase 3 study (174‐10 969 ng/mL).^14^ Within the plasma isavuconazole concentration range achieved in the pivotal phase 3 study, previous reported exposure‐response analyses suggested no exposure‐response relationship for efficacy or safety.^14^, ^15^ However, as no efficacy, safety, or pharmacokinetic data are available in a Japanese patient population to date, it is not currently possible to determine whether a dose adjustment is required. After data from the phase 3 study in adult Japanese patients with deep mycosis are available, further discussion can be had.

In the present study, it was confirmed that single administration of up to 400‐mg equivalent of isavuconazole and multiple doses of 200‐mg equivalent of isavuconazole were safe and well tolerated in healthy Japanese subjects. There were no serious adverse events, and no subjects were withdrawn from the study due to TEAEs. A total of 21 TEAEs were reported, all of which were mild. No clinically relevant changes in vital signs or ECG were observed. Abnormal laboratory values were infrequent.

## Conclusion

This study showed that CL was lower in healthy Japanese subjects than that previously reported in predominantly White populations and was similar to CL in non‐Japanese Asian populations. The exact reason for the difference in CL has not been established. Although lower CL was seen in the present study, the range of the plasma isavuconazole concentration in this study was within the range of the pivotal phase 3 study, where no relationship between isavuconazole exposure and either efficacy or safety was found. After data in adult Japanese patients with deep mycosis are available, further discussion can be had. It was confirmed that single administration of up to 400‐mg equivalent of isavuconazole and multiple doses of 200‐mg equivalent of isavuconazole were safe and well tolerated in healthy Japanese subjects.

## Conflicts of Interest

S.S. and A.O. are employees of Asahi Kasei Pharma, the sponsor of this study. Y.K. received a consulting fee from Asahi Kasei Pharma.

## Supporting information

Figure S1. Mean plasma isavuconazole concentration–time profile after single administration of isavuconazonium sulfate by dose level. (a, b) Mean plasma concentration–time profile after single oral or IV administration of isavuconazonium sulfate of 100‐mg equivalent of isavuconazole at 24 hours (a) and up to 480 hours (b). (c, d) Mean plasma concentration–time profile after single oral or IV administration of isavuconazonium sulfate of 200‐mg equivalent of isavuconazole at 24 hours (c) and up to 480 hours (d). (e, f) Mean plasma concentration–time profile after single oral or IV administration of 400 mg equivalent of isavuconazole at 24 hours (e) and up to 480 hours (f). Each value below the limit of quantification was set at 0 in the calculation of mean values. PO 100 mg, oral administration of 100‐mg equivalent of isavuconazole; PO 200 mg, oral administration of 200‐mg equivalent of isavuconazole; PO 400 mg, oral administration of 400‐mg equivalent of isavuconazole; IV 100 mg, IV administration of 100‐mg equivalent of isavuconazole; IV 200 mg, IV administration of 200 mg equivalent of isavuconazole; IV 400 mg, IV administration of 400‐mg equivalent of isavuconazole. IV, intravenous; PO, oral.

Supporting Information, Additional supplemental information can be found by clicking the Supplements link in the PDF toolbar or the Supplemental Information section at the end of the web‐based version of this article.
