# Supplementary material for: Pharmacokinetics, Safety, and Tolerability of Single and Multiple Doses of Isavuconazonium Sulfate in Healthy Adult Japanese Subjects
Source: Clin Pharmacol Drug Dev. 2022 Feb 21;11(6):744–53. doi: 10.1002/cpdd.1079 (PMC9303187; doi:10.1002/cpdd.1079)
Supplement: Supplementary file 1 — Figure S1. Mean plasma isavuconazole concentration–time profile after single administration of isavuconazonium sulfate by dose level. (a, b) Mean plasma concentration–time profile after single oral or IV administration of isavuconazonium sulfate of 100‐mg equivalent of isavuconazole at 24 hours (a) and up to 480 hours (b). (c, d) Mean plasma concentration–time profile after single oral or IV administration of isavuconazonium sulfate of 200‐mg equivalent of isavuconazole at 24 hours (c) and up to 480 hours (d). (e, f) Mean plasma concentration–time profile after single oral or IV administration of 400 mg equivalent of isavuconazole at 24 hours (e) and up to 480 hours (f). Each value below the limit of quantification was set at 0 in the calculation of mean values. PO 100 mg, oral administration of 100‐mg equivalent of isavuconazole; PO 200 mg, oral administration of 200‐mg equivalent of isavuconazole; PO 400 mg, oral administration of 400‐mg equivalent of isavuconazole; IV 100 mg, IV administration of 100‐mg equivalent of isavuconazole; IV 200 mg, IV administration of 200 mg equivalent of isavuconazole; IV 400 mg, IV administration of 400‐mg equivalent of isavuconazole. IV, intravenous; PO, oral. [file CPDD-11-744-s002.docx]

Supplemental Figure 1. Mean plasma isavuconazole concentration-time profile after single administration of isavuconazonium sulfate by dose level.
