# Supplementary figures and images for: Pharmacokinetics, Safety, and Tolerability of Single and Multiple Doses of Isavuconazonium Sulfate in Healthy Adult Japanese Subjects
Source: Clin Pharmacol Drug Dev. 2022 Feb 21;11(6):744–53. doi: 10.1002/cpdd.1079 (PMC9303187; doi:10.1002/cpdd.1079)

**A**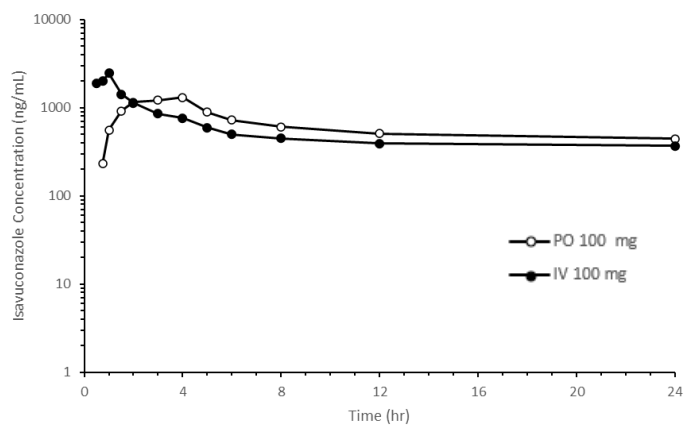**B**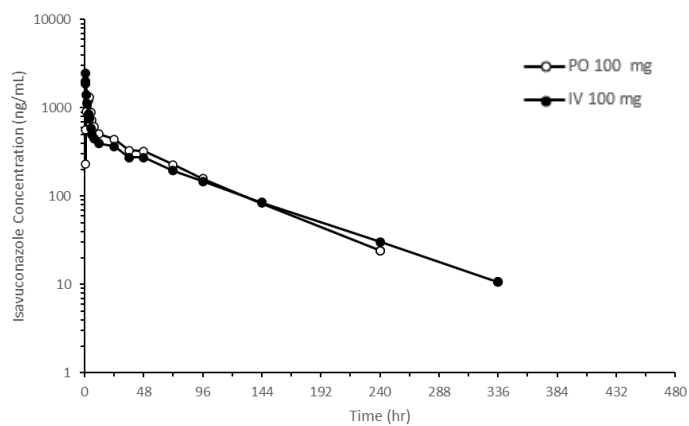**C**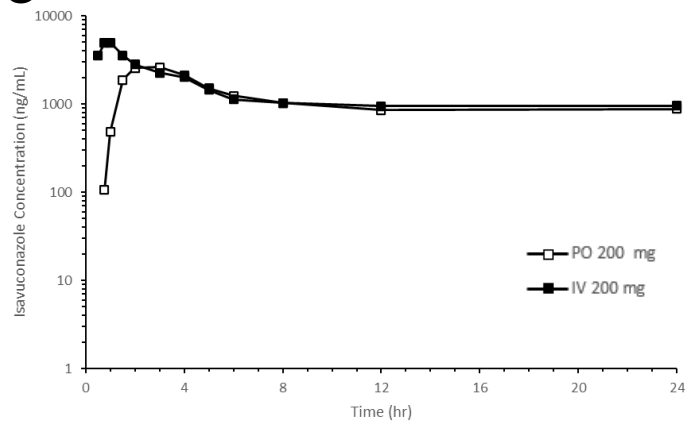**D**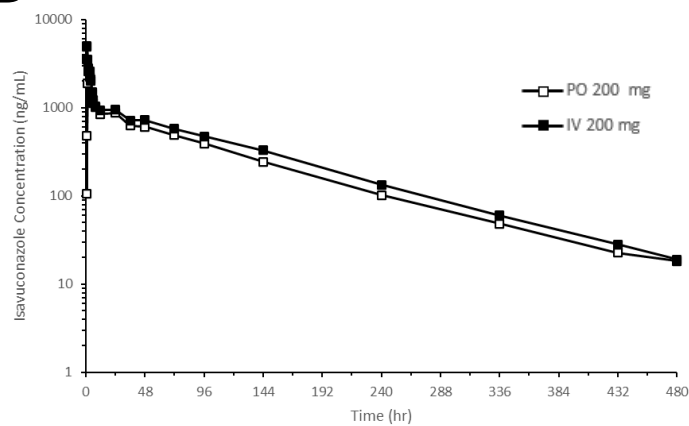**E**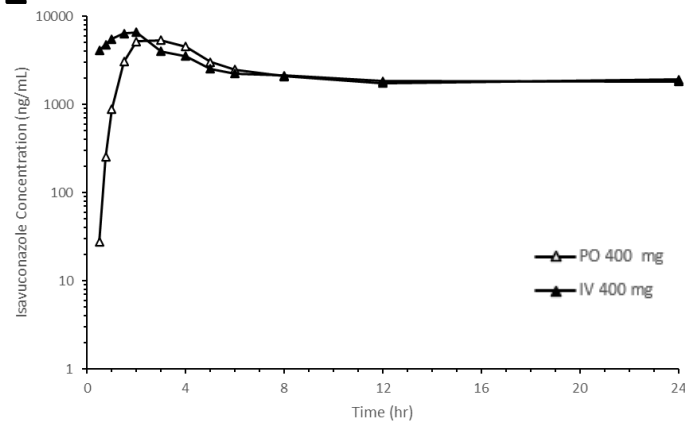**F**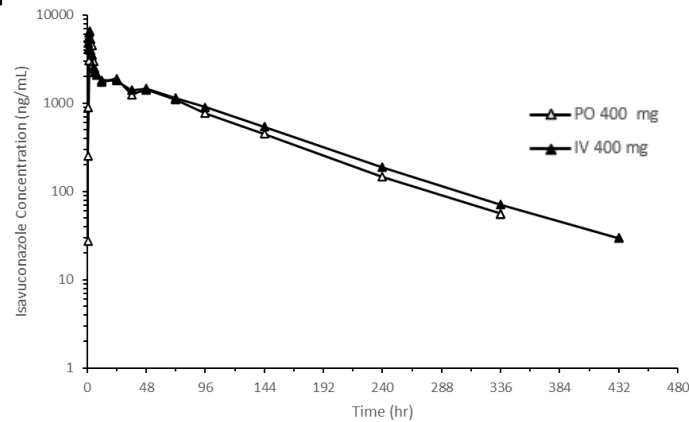

Supplement: Supplementary file 2 — Supporting Information, Additional supplemental information can be found by clicking the Supplements link in the PDF toolbar or the Supplemental Information section at the end of the web‐based version of this article. [file CPDD-11-744-s001.pdf]
